# Aberrant Pulmonary Vascular Growth and Remodeling in Bronchopulmonary Dysplasia

**DOI:** 10.3389/fmed.2016.00021

**Published:** 2016-05-20

**Authors:** Cristina M. Alvira

**Affiliations:** ^1^Department of Pediatrics, Division of Critical Care Medicine, Stanford University School of Medicine, Stanford, CA, USA

**Keywords:** pulmonary angiogenesis, pulmonary hypertension, alveolarization, chronic lung disease, VEGF, HIF, nitric oxide

## Abstract

In contrast to many other organs, a significant portion of lung development occurs after birth during alveolarization, thus rendering the lung highly susceptible to injuries that may disrupt this developmental process. Premature birth heightens this susceptibility, with many premature infants developing the chronic lung disease, bronchopulmonary dysplasia (BPD), a disease characterized by arrested alveolarization. Over the past decade, tremendous progress has been made in the elucidation of mechanisms that promote postnatal lung development, including extensive data suggesting that impaired pulmonary angiogenesis contributes to the pathogenesis of BPD. Moreover, in addition to impaired vascular growth, patients with BPD also frequently demonstrate alterations in pulmonary vascular remodeling and tone, increasing the risk for persistent hypoxemia and the development of pulmonary hypertension. In this review, an overview of normal lung development will be presented, and the pathologic features of arrested development observed in BPD will be described, with a specific emphasis on the pulmonary vascular abnormalities. Key pathways that promote normal pulmonary vascular development will be reviewed, and the experimental and clinical evidence demonstrating alterations of these essential pathways in BPD summarized.

## Introduction

A significant portion of lung development occurs after birth during the alveolar stage of development. During this final stage, the alveolar ducts divide into alveolar sacs by secondary septation, and the pulmonary capillary bed expands *via* angiogenesis to markedly increase the gas exchange surface area of the lung (1). However, postnatal completion of growth renders the lung highly susceptible to insults that disrupt this developmental program. This is particularly evident in the setting of preterm birth, where disruption of alveolarization causes bronchopulmonary dysplasia (BPD), the most common complication of prematurity (2). While advances in the supportive care of extremely premature infants have reduced mortality, the morbidities associated with severe BPD persist (3). Accompanying this increase in survival, the clinical and pathologic features of BPD have changed significantly. In contrast to the severe lung injury characterizing “old BPD” as originally described by Northway (4), premature birth earlier in gestation appears to disrupt the normal program of alveolar and vascular development, resulting in the “new BPD,” characterized by an arrest in alveolar and vascular development (5).

The impaired pulmonary angiogenesis observed in patients with BPD appears to be the key to the pathogenesis. Proangiogenic factors are decreased in the lungs of infants dying from BPD (6) and in animal models of BPD induced by hyperoxia (7). Administration of anti-angiogenic agents to neonatal rats impairs both pulmonary angiogenesis and alveolarization (8, 9), and overexpression of proangiogenic factors, such as vascular endothelial growth factor (VEGF), rescues the adverse effects of hyperoxia on alveolarization (7). Moreover, in addition to simple decreases in pulmonary microvascular growth, the pulmonary vascular abnormalities in BPD may also include pathologic remodeling and heightened tone, leading to the development of pulmonary hypertension (PH), as well as an increase in the development of abnormal aorto–pulmonary communications, potentially promoting intrapulmonary shunting.

This review presents an overview of lung development and details the pathology of the “new” BPD, characterized by an arrest in normal lung development. Specific focus will be centered upon the pulmonary vascular abnormalities in BPD including impaired pulmonary angiogenesis, abnormal pulmonary vascular remodeling, heightened pulmonary vascular tone, and development of abnormal collateral circulations. Key pathways that promote normal pulmonary vascular development will be reviewed, and the experimental and clinical evidence demonstrating how these pathways are altered in BPD summarized.

## Overview of Normal Airway and Pulmonary Vascular Development

Lung development begins when the primitive lung bud emerges from the ventral foregut and divides during the embryonic stage of development (4–7 weeks gestation), forming two lung buds lying on either side of the future esophagus and surrounded by splanchnic mesenchyme (10). The remaining four stages follow sequentially, beginning with the development of the pre-acinar airways *via* branching morphogenesis during the pseudoglandular stage (7–17 weeks gestation). During the canalicular stage (17–25 weeks gestation), the airways divide further to form the alveolar ducts, and the distal lung mesenchyme thins to allow close approximation of the developing respiratory epithelium and vascular endothelium. Widening and branching of these distal air sacs occurs in the saccular stage (26–36 weeks gestation), and finally, during the alveolar stage (36 weeks gestation onward), the terminal alveoli form by the process of secondary septation and rapidly increase in number throughout early childhood (11).

The mature lung contains approximately 500 million alveoli (12), each surrounded by a network of pulmonary capillaries allowing close proximity of the air filled alveolus with the blood-filled capillary. This intimate association of the pulmonary microcirculation with the terminal airspaces is imperative for efficient gas exchange. Therefore, the pulmonary blood supply must develop in close relationship to the airways throughout lung development (10). Early recognition that the branching of the pre-acinar arteries (formed by the end of the pseudoglandular stage) occurs at the same time and along a similar pattern, as the branching of the airways, suggested that the airways may provide a template for the development of the pulmonary arteries and veins (13).

The pulmonary circulation likely forms through a combination of vasculogenesis, the *de novo* formation of vessels from the differentiation of primitive angioblasts and hemangioblasts, and angiogenesis, the sprouting and branching of new vessels from existing vessels (14, 15). However, the degree to which each process contributes to the formation of the pulmonary vasculature at each stage of development remains a source for debate. Early evidence supported the notion that the proximal arteries form by angiogenic sprouting from the main pulmonary trunk and that distal branches form *de novo* in the distal mesenchyme *via* vasculogenesis. Using a method to make a cast of the developing pulmonary vasculature in fetal rats (from E9 to E20), deMelo et al. showed that isolated “blood lakes” form in the periphery of the lung (presumably by vasculogenesis) as early as E9. This was followed by the central sprouting of the proximal arteries, with the formation of five to seven generations of branching by E14, and connections between the proximal and distal vessels by E13–14 (16). In contrast, using transgenic reporter mice that express LacZ under the control of an endothelial specific promoter, Schachtner et al. found evidence of connections between the proximal, branching pulmonary arteries, and endothelial cells located in the distal mesenchyme as early as E10.5, several days before patency of the central pulmonary arteries has occurred. These findings suggested the authors that vasculogenesis may contribute to the development of the proximal pulmonary vasculature as well (17).

Prior to term birth, the density of the peripheral pulmonary vessels markedly increases in density, suggesting expansion of the capillary network by angiogenesis (16). After birth, the pulmonary capillary network continues to expand, resulting in a 35-fold increase by adulthood (13). Airway and vascular development are closely linked, with the disruption of one process impairing the other, and each culminating in a global disruption of lung development (18). Moreover, pulmonary vascular development continues throughout all stages of lung development in a manner proportional to the overall growth of the lung, rendering it vulnerable to perturbations occurring in both embryonic and postnatal life (17).

## Extreme Lung Immaturity and Arrested Lung Development: The “New” BPD

In 1967, Northway et al. used the term BPD to describe a novel form of chronic lung disease that developed in preterm infants (mean gestational age of 32 weeks) who had a history of neonatal respiratory distress (4). This original form of BPD was associated with positive-pressure ventilation and prolonged oxygen therapy, and characterized by histologic evidence of severe lung injury (e.g., inflammation, protein-rich edema, airway epithelial metaplasia, and peribronchial fibrosis) and marked airway and pulmonary vascular smooth muscle hypertrophy (19, 20). Abnormalities in the pulmonary vasculature were also a feature of the disease. Pathologic examination of post-mortem lung tissue from a small group of infants with BPD who survived for at least 1 month demonstrated decreased density of peripheral pulmonary arteries as compared to control patients, both by barium angiogram and histologic measures (21).

However, advances in medical therapy, including antenatal steroids, surfactant replacement therapy, and the institution of lung protective strategies of ventilation, have permitted the survival of extremely immature, very low birth weight (VLBW) infants. Accompanying this increase in survival, the clinical, radiographic, and pathological features of BPD have changed significantly. In contradistinction to the original form of BPD, birth of VLBW infants during the late canalicular or early saccular stages of lung development appears to disrupt the normal alveolar and vascular development, resulting in the “new BPD.” Margraf et al. described the lung pathology of this new, post-surfactant form of BPD in a small case series of infants who died with severe BPD. One of the most striking findings observed by the authors was the severely reduced alveolar number in the infants with BPD compared to controls, with little evidence of the normal, physiologic increases in alveolar number typically observed with advancing age (22). Similarly, Husain et al. also showed evidence of arrested acinar development in a series of infants with post-surfactant BPD, including both reductions in acinar number and increases in acinar size (23).

Pathologic data obtained from autopsy specimens can be difficult to interpret and generalize to the entire disease population, as these samples often represent the most severe lung disease in patients with BPD (24). This is particularly true now that key advances in the medical care of preterm infants have markedly decreased mortality, such that infants who die from BPD in this era truly represent an extremely ill subset of patients. However, Coalson et al. obtained important information surrounding the evolving histopathology in infants with this “new” form of BPD in a small series that examined open lung biopsies from low-birth weight babies on ventilator support who received surfactant but not steroids. Those infants also demonstrated alveolar simplification but minimal metaplasia, and variable degrees of inflammation and abnormal extracellular matrix deposition (25).

## Abnormalities in Pulmonary Vascular Development and Remodeling

### Dysmorphic Pulmonary Microvascular Development

In addition to alveolar simplification (i.e., decreased complexity of distal lung septation), the pathology of this “new” form of BPD also appears to include abnormalities in the development of the pulmonary microvasculature. A comparison of autopsy specimens taken from infants dying from BPD compared to infants dying without lung disease at similar post-conceptional ages demonstrated that the lungs of infants with BPD had an overall reduction in immunostaining for the endothelial specific marker CD31, suggesting a decrease in pulmonary microvascular density. Moreover, the pulmonary capillaries, when present, appeared to be abnormally dilated and frequently located within thickened alveolar septa, rather than immediately adjacent to the alveolar epithelium (6). These reductions in the growth of the distal pulmonary vasculature were in keeping with the pathologic findings observed in specimens obtained from patients dying of BPD in the pre-surfactant era, where decreases in arterial number and cross-sectional area were thought to contribute to the increased dead space ventilation observed in those infants (26).

However, additional studies have suggested that rather than a simple decrease in pulmonary vascular growth, the vascular abnormalities observed in patients with BPD might be more accurately described as “dysmorphic.” In the open lung biopsy samples obtained by Coalson et al., evidence of abnormal capillary development was apparent, with CD31 immunostaining demonstrating an “adaptive dysmorphic pattern of vascular organization.” This pattern included a paucity of capillaries within the walls of the thinned abnormally enlarged alveoli, and dilated, more abundant capillaries in other sites (25). In contrast, a stereology-based assessment of endothelial cell volume in short- and long-term ventilated preterm infants demonstrated that total endothelial cell volume increased in ventilated infants as compared to age-matched controls, in association with an increase in total parenchymal volume, suggesting an expansion of the pulmonary microvasculature. However, in the long-term ventilated patients, the capillary network was simplified, had decreased branching, and retained the dual capillary pattern characteristic of the saccular lung, features predicted to decrease gas exchange efficiency (27). Taken together, these studies suggest that variable abnormalities in the pulmonary capillaries may be observed in BPD, with suppressed vascular growth at some stages of the disease, and excessive, dysmorphic growth at other stages, perhaps representing a maladaptive compensatory response.

### Abnormal Muscularization, Heightened Vascular Tone, and the Development of Pulmonary Hypertension

In his original description of BPD, Northway noted that some patients had evidence of medial hypertrophy of the pulmonary arteries, suggesting the development of PH (4). This histologic finding was confirmed by clinical studies demonstrating elevations in pulmonary arterial pressures (PAPs) and pulmonary vascular resistance (PVR) by either cardiac catheterization or enchocardiography in survivors of BPD. In one such study, Fouron et al. found that the majority of patients with BPD in the “acute phase” had echocardiographic evidence of PH, and that pulmonary pressures remained high in those infants who eventually died, but normalized in infants who recovered (28). However, long-term follow-up of patients with pre-surfactant BPD and PH showed that in many patients, elevations in PAPs persisted through early childhood (29).

**Figure 1:**
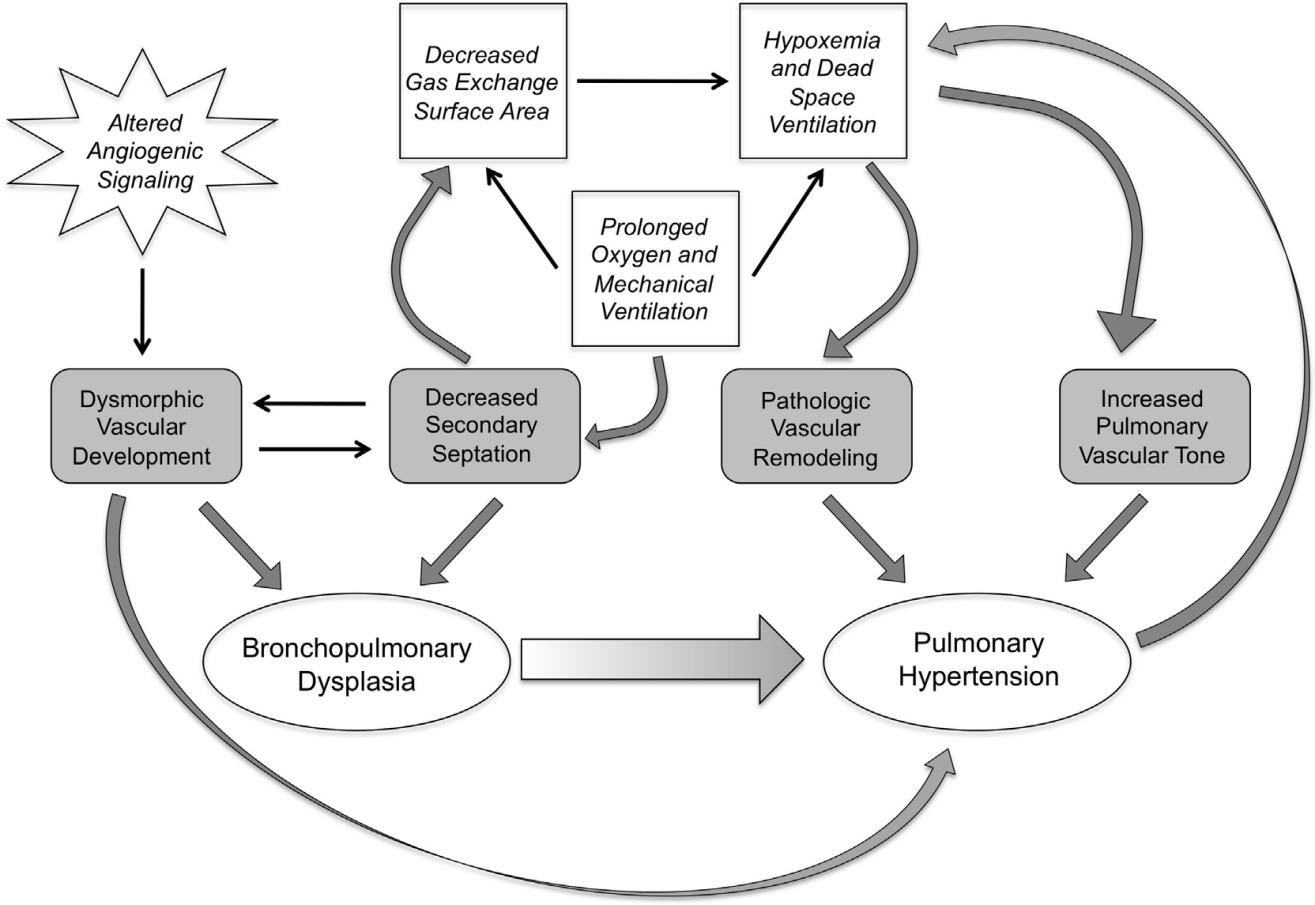
**The interplay of pathologic and clinical factors that lead to pulmonary hypertension in bronchopulmonary dysplasia**. Dysmorphic vascular development as a result of altered angiogenic signaling combines with impairments in secondary septation, leading to the development of BPD. These events set the stage, pre- and postnatally, for the development of pulmonary hypertension. The decrease in gas exchange surface area resulting from the impaired secondary septation also sets up a vicious cycle of hypoxemia and dead space ventilation that prolongs the need for mechanical ventilation and oxygen therapy, and induces pathologic changes in pulmonary vascular remodeling and tone, further increasing the risk of pulmonary hypertension.

With the evolution of BPD in the post-surfactant era, the development of PH remains a significant feature of the disease for a subgroup of patients and significantly impacts long-term prognosis. In a prospective study of preterm infants using a broad echocardiogram-based definition of PH, early evidence of PH was found in more than 40% of patients at 7 days of age, and late PH found in almost 15% of patients at 36 weeks PMA. In patients who develop severe BPD, the incidence of late PH appears to be significantly higher ranging from 30 to 50% of patients (30–32). Moreover, the presence of PH in patients with BPD is independently associated with a greater increase in the odds of death (30, 32), with mortality rates as high as 40% in some studies (33). Of note, the risk of death appears to be highest in the first 6 months after the diagnosis of PH, and the majority of infants with BPD and PH who survive beyond a mean of 10 months of age demonstrate an improvement in the severity of PH (33). Numerous risk factors have been associated with an increased incidence of PH in patients with BPD including: oligohydramnios (32, 34), low apgar scores (32, 34), postnatal sepsis (34), small for gestational age (33), and prolonged use of positive-pressure ventilation (31). Of note, while the risk of developing PH is significantly higher in patients with severe versus moderate BPD (32), a smaller percentage of infants with no, mild, or moderate BPD also develop late PH. This suggests that the risk for developing late PH may not be primarily dictated by the severity of lung disease (31). In addition, early PH appears to predict the development of BPD (35), again highlighting the link between abnormalities in the pulmonary circulation and impairments in distal lung development (Figure 1). While a complete understanding of the mechanisms leading to PH in a subset of patients is lacking, the data suggest that patients with BPD and PH demonstrate abnormalities in both distal pulmonary artery muscularization and tone.

#### Abnormalities in Pulmonary Arterial Muscularization

In his original report, Northway et al. identified “early vascular lesions of the pulmonary hypertensive type” in the cohort of infants in the later stages of the disease, which comprised medial hypertrophy and characteristic breakdown of the elastic lamina (4). Later studies demonstrated similar pathologic remodeling of small pulmonary arteries in patients with the pre-surfactant form of BPD. In a small study of preterm infants with severe BPD and cor pulmonale, affected patients demonstrated an increase in the percent medial thickness of distal arteries and an extension of arterial smooth muscle into peripheral arteries such that the majority of alveolar wall arteries were completely muscularized (26). Further, abnormal muscularization of the pulmonary arteries was often a feature of pre-surfactant BPD even in patients that did not develop cor pulmonale. In premature infants with respiratory distress syndrome (RDS) who died early in life while still requiring mechanical support, many demonstrated increased medial thickness of distal arteries, appearing similar to the muscularized small arteries characteristic of a term infant on the first day of life (20). Moreover, in keeping with the findings of Bush et al., those infants with BPD who developed cor pulmonale had evidence of marked muscularization of small arteries, with complete muscularization of arteriolar wall arteries, and some patients with intimal proliferation of larger arteries (20). In a separate study, the combination of abnormal muscularization of distal arteries with variable degrees of either increased or decreased pulmonary capillary density suggested a “dual process of adaptation and response to injury in a hypoplastic lung” (36).

In the post-surfactant era, mortality of premature infants has decreased, thus limiting the availability of autopsy specimens that would allow careful characterization of the pulmonary arterial histopathologic changes in the “new” form of BPD. However, in at least one study, it appears that the abnormal muscularization of peripheral arteries remains a consistent pathologic feature. In a study of post-mortem tissue obtained from surfactant-treated preterm infants with BPD, there was evidence of increased arterial wall thickness and muscularization of distal vessels in preterm infants with severe BPD, although these histologic changes were less marked than those observed in specimens obtained from infants who developed PH in the setting of persistent pulmonary hypertension of the newborn (PPHN) and premature rupture of membranes (PROM) (37). While the mechanisms that specifically induce pathologic pulmonary vascular remodeling in BPD are unknown, they are hypothesized to include some of the well recognized injurious stimuli that disrupt distal lung growth including hyperoxia, mechanical ventilation, and inflammation (38).

#### Abnormalities in Pulmonary Vascular Tone

In addition to abnormal pulmonary arterial remodeling, heightened pulmonary arterial tone also appeared to be an important component of the PH observed in patients with pre-surfactant BPD. Survivors of BPD with persistent oxygen requirements and evidence of right ventricular hypertrophy (RVH) on ECG had evidence of PH on cardiac catheterization, with pulmonary vascular beds that were responsive to even low levels of oxygen (39). In a prospective study of 15 patients with moderate to severe BPD and PH undergoing cardiac catheterization, all patients demonstrated a reduction in PA pressure with supplemental oxygen, and variable responses to vasodilator therapy depending on the presence or absence of systemic–pulmonary collaterals (40).

Elevated pulmonary vascular tone remains a key feature of the PH in BPD survivors in the post-surfactant era. A study examining BPD survivors with PH who underwent cardiac catheterization found that most patients have significant pulmonary vascular reactivity, demonstrating elevations in mean PAP with hypoxia, and conversely, decreased mean PAP with the combination of hyperoxia and inhaled nitric oxide (iNO) (41). Similarly, in a study reporting data from the cardiac catheterization of 13 patients with BPD and PH, PAP and PVR decreased significantly with vasodilator therapy (100% O_2_ or iNO) in the majority of patients, but still remained elevated above normal levels (33).

### Abnormal Collateral Circulations

In an early report, cardiac catheterization of two premature infants who required prolonged mechanical ventilation found that although these infants had normal pulmonary pressures, they both had evidence of large systemic collaterals with left to right shunts, a finding the authors hypothesized likely contributed to their persistent ventilator dependence (42). This report was followed by the description of similar collateral vessels in a subgroup of patients with severe BPD and PH, in whom the administration of vasodilators had deleterious results, inducing respiratory acidosis, pulmonary edema, and more severe hypoxemia (40). However, it was not clear from either report whether these abnormal vessels were congenital in nature or acquired, resulting from persistent hypoxemia, pathologic alterations in pulmonary blood flow, or disrupted lung development (43).

More recently, histologic examination of lung tissue from a number of patients dying with severe BPD demonstrated the presence of numerous smaller intrapulmonary arteriovenous anastomotic vessels (IAAV) that appear similar to the “misalignment of veins” seen in alveolar capillary dysplasia. These vascular channels are located in the lobar periphery and extend toward the pulmonary arteries, appearing to connect with the microvascular plexus surrounding the pulmonary arteries and airways (44). Of note, these intrapulmonary anastomotic vessels are not unique to BPD, but observed in other diseases of impaired alveolarization. For example, in a similar study examining the lung tissue of infants dying from severe congenital diaphragmatic hernia (CDH) and associated PH, the lungs of all patients demonstrated prominent, engorged intrapulmonary vessels connecting the pulmonary veins to the microvessels surrounding the pulmonary arteries (45). Similar, intrapulmonary, bronchopulmonary anastomoses have also been noted in infants dying from meconium aspiration syndrome (46). These prominent IAAV may represent the failure of the normal fetal IAAV circulation to close after birth and have the potential to permit right to left intrapulmonary shunting, thus contributing to the hypoxemia observed in patients with severe BPD.

## Key Pathways Directing Normal Pulmonary Vascular Development and Function

Extensive clinical evidence obtained from patients with BPD in both the pre- and post-surfactant era has identified impaired and dysmorphic pulmonary vascular development as a key feature of the disease. These data suggest that the normal pathways that promote postnatal pulmonary vascular growth are disrupted in BPD. In this section, a number of key pathways that direct normal pulmonary vascular growth and function will be reviewed, and evidence demonstrating alterations in these pathways in experimental models of BPD (Table 1) and clinical studies will be summarized.

**Table 1:** **Molecular mechanisms contributing to impaired alveolar and pulmonary vascular growth in animal models**.

Molecule	Physiologic functions and disruption in animal models of BPD	Reference
VEGF	Global deletion delays endothelial cell differentiation, impairs vascular development, and induces lethality at E8.5	(55, 56)
	Isoform-specific deletion (VEGF_164_ and VEGF_188_) impairs lung microvascular development and delays airspace maturation	(57)
	Postnatal inhibition decreases somatic growth and impairs alveolarization	(58)
	Decreased expression in response to hyperoxia and mechanical ventilation in numerous animal models	(60–64)
	Overexpression promotes lung angiogenesis, and inhibits hyperoxia-induced alveolar simplification and mortality in rats	(66)
FLK-1	Homozygous deletion prevents endothelial cell differentiation and blood vessel formation, and induces embryonic lethality	(53)
	Decreased expression in response to mechanical ventilation in neonatal mice	(63, 64)
	Postnatal inhibition impairs lung angiogenesis and alveolarization and induces pulmonary hypertension in neonatal rats	(8, 9)
FLT-1	Homozygous deletion causes disorganization of vascular development and induces embryonic lethality	(54)
	Decreased expression in response to mechanical ventilation in preterm baboons	(63)
NFκB	Pharmacologic inhibition in neonatal mice impairs lung angiogenesis and alveolarization and decreases *Flk-1* expression, and exaggerates the impairment in angiogenesis and alveolarization induced by systemic endotoxin	(65, 66)
HIF-1α	Global deletion results in numerous cardiac and vascular abnormalities and embryonic lethality at E10.5	(71, 72)
	Decreased expression in response to mechanical ventilation in preterm baboons and lambs	(69, 75)
	Stabilization of HIF improves alveolar growth in preterm baboons and neonatal rats exposed to combined endotoxin/hyperoxia	(78–80)
HIF-2α	Global deletion results in perinatal mortality due to respiratory failure, decreased VEGF expression, and decreased surfactant	(73)
	Decreased expression in mechanical ventilation of preterm baboons and lambs, and in neonatal rats exposed to chronic hypoxia	(69, 75)
NO/eNOS	Deletion of eNOS impairs VEGF-mediated angiogenesis and neovascularization, worsens pulmonary hypertension in adult mice exposed to chronic hypoxia, and increases susceptibility of neonatal mice to the impaired alveolarization induced by hyperoxia	(88, 90, 91, 99, 100)
	Decreased eNOS expression in mechanically ventilated preterm baboons and lambs, in fetal lambs exposed to intrauterine endotoxin	(94–96)
	Decreased NO production in pulmonary arteries from fetal lambs with intrauterine growth restriction	(97)
H_2_S	Deletion of enzymes that produce H_2_S impairs alveolarization, decrease lung vascular growth, and induce pathologic vascular remodeling	(104)
	Exogenous administration improves alveolarization, limits pulmonary hypertension, and decreases lung inflammation in neonatal rats and mice exposed to hyperoxia	(105, 106)
Retinoic acid	Deletion of the RA receptor-gamma impairs alveolarization and decreases lung elastin	(110)
	Promotes alveolar regeneration in adult mice with elastase-induced emphysema and limits the impaired alveolarization induced by glucocorticoids in neonatal mice	(108, 109)
LPA	Deletion of the LPA-receptor 1 limits lung inflammation and fibrosis, and improves survival in neonatal rats exposed to hyperoxia	(116)
	Pharmacologic blockade of LPA receptors -1 and -3 limits pulmonary hypertension in newborn rats exposed to hyperoxia	(116)
EC-SOD	Deletion impairs alveolarization and lung angiogenesis, and decreases FLK-1 protein expression in neonatal mice	(118)
	Alveolar epithelial overexpression preserves alveolar and vascular growth of neonatal mice exposed to hyperoxia	(119)

### Vascular Endothelial Growth Factor

The endothelial cell mitogen and survival factor, VEGF, is essential for normal blood vessel development. Alternate splicing from a single gene produces three distinct isoforms: VEGF_120_, VEGF_164_, and VEGF_188_. These three isoforms demonstrate differential binding to heparin sulfate and affinities for the two predominant receptors: tyrosine kinases fms-like-tyrosine kinase-1 (FLT-1) and fetal liver kinase-1 (FLK-1) (47). The lung expression of the two heparin-binding isoforms, VEGF_164_ and VEGF_188_, increases during the late saccular stage of development in the mouse and remains high through adulthood (47), with VEGF_188_ becoming the predominant isoform by late alveolarization (48). Paralleling the expression pattern observed with VEGF, FLT-1 and FLK-1 are highly expressed by endothelial cells during lung development (49), and in the murine lung, the expression of both receptors increase during alveolarization and remain high in the adult lung (47, 48). In addition to the full-length, membrane-bound form of FLT-1, a soluble form, comprised of the extracellular ligand binding domain, can be produced by alternative splicing from a single gene transcript (50, 51). It is thought that this soluble form (sFLT-1) may function as a physiologic inhibitor of angiogenesis given its ability to sequester VEGF ligands and prevent them from binding to the active transmembrane receptors (52).

The absolute requirement of intact VEGF signaling for vascular development is underscored by the severe phenotypes observed in mice containing targeted disruptions of discrete components of the pathway. Homozygous deletion of *Flk-1* in mice results in early embryonic lethality, complete absence of blood vessel formation, and a failure of endothelial differentiation (53). In contrast, while homozygous deletion of *Flt-1* also results in embryonic lethality, endothelial cell differentiation is preserved, and the vasculature develops but is very disorganized (54). Targeted deletion of *Vegf* in mice delays endothelial cell differentiation and severely impairs vascular development, resulting in embryonic lethality between E8.5 and 9.5 (55). Of note, even the absence of a single allele of *Vegf* impairs vascular development and induces embryonic lethality (56). Absence of the two heparin-bound isomers, VEGF_164_ and VEGF_188_, impairs lung microvascular development and delays airspace maturation in mice, suggesting that these isoforms which are bound tightly in the extracellular matrix may provide a source of local VEGF specifically essential for pulmonary vascular development (57).

In addition to these indispensable roles for VEGF during embryonic development, VEGF is also an important mediator of postnatal organ growth and development. Partial inhibition of VEGF in mice during the first week of life using an inducible gene targeting strategy decreases somatic growth and impairs organ development, while complete inhibition by the administration of a soluble VEGF receptor chimeric protein exaggerates these effects on organ development and growth and specifically impairs alveolarization (58). Moreover, the spatial expression of VEGF during late development is critical. Expression of VEGF_164_ in the alveolar type II (ATII) cells using the SP-C promoter induces earlier and higher levels of VEGF in the developing lung and increases pulmonary blood vessel growth, but disrupts branching morphogenesis and inhibits alveolar type I cell differentiation (59). Taken together, these studies demonstrate the importance of tightly regulated temporal and spatial expression of VEGF for normal vascular development.

Abnormalities in VEGF signaling appear to be a key mechanism in the impaired alveolarization and angiogenesis observed in experimental models of BPD. Chronic exposure to hyperoxia in neonatal rabbits decreases VEGF gene and protein expression by alveolar epithelial cells (60). In neonatal rats, high levels of hyperoxia decrease *Vegf* gene expression (61), and sustained hyperoxia from postnatal day (P)4–14 impairs alveolarization, and suppresses *Vegf* and *Hif-2α* gene expression and VEGF receptor protein expression (61, 62). In the preterm baboon model of BPD, mechanical ventilation and oxygen reduce pulmonary capillary volume, impair alveolarization, and repress the physiologic increase in VEGF and FLT-1 observed in control animals (63). Similarly, mechanical ventilation of neonatal mice during the late saccular stage of development induces alveolar simplification and reduces lung expression of VEGF and FLK-1 (64). Inhibiting constitutive activation of nuclear factor-κB, a direct regulator of *Flk-1* during alveolarization, impairs pulmonary angiogenesis and disrupts alveolarization in neonatal mice (65), and exaggerates the impairment in angiogenesis and alveolarization induced by systemic endotoxin (66). Moreover, blocking angiogenesis in neonatal rats directly using either non-specific anti-angiogenic compounds, or a selective FLK-1 inhibitor, decreases pulmonary arterial density and impairs alveolarization, thus providing some of the first direct, experimental evidence to support the notion that angiogenesis actively promotes distal lung growth (8). In fact, even the administration of a single dose of the FLK-1 inhibitor significantly decreases pulmonary arterial density, impairs alveolarization, and induces pulmonary artery muscularization and RVH that persist into adulthood (9). Consistent with these studies, overexpression of VEGF in newborn rats is effective in increasing survival, promoting lung angiogenesis, and preventing hyperoxia-induced alveolar simplification (67).

### Hypoxia-Inducible Factor

Fetal development occurs at low oxygen tension. The hypoxia-inducible factor (HIF) family of transcription factors is a key regulator of O_2_ homeostasis, activating genes critical for energy metabolism, oxygen transport, and angiogenesis. The HIFs are heterodimeric transcription factors comprised of oxygen sensitive subunits (HIF-1α, HIF-2α, and HIF-3) paired with the constitutively expressed HIF-1β (previously known as ARNT) subunit. Under normal oxygen tension, the O_2_ sensitive subunits are continuously degraded. However, under conditions of low oxygen tension, HIF degradation is inhibited, resulting in HIF protein stabilization and accumulation, thereby promoting the binding of HIF to hypoxia-response elements (HREs) located within the promoters of downstream target genes, including *VEGF*. During lung development, HIF-1α is expressed in the branching epithelium, and HIF-2 expressed in both the epithelium and the mesenchyme (68). In the primate lung, expression of both HIF-1α and HIF-2 is high in the third trimester of pregnancy; however, at term birth, HIF-2 expression remains high while HIF-1α is absent (69). In mouse lung, HIF-2α expression also increases immediately after birth and remains high throughout alveolarization, with production predominantly by ATII cells and colocalizing with VEGF expression (70).

The importance of this pathway in vascular development was highlighted by studies that performed targeted deletions of HIF family members in mice. Loss of *Hif-1α* results in embryonic lethality at E10.5, with null embryos demonstrating numerous cardiac and vascular malformations including vascular regression and abnormal vascular remodeling (71, 72). Interestingly, although this phenotype was similar to that seen in the VEGF null mice, *Hif-1α*^−/−^ mice were found to have normal levels of *Vegf* mRNA, suggesting that the vascular malformations observed were independent of impairments in VEGF expression. In contrast, *Hif-2α*^−/−^ mice die from RDS during the perinatal period in association with decreases in ATII-mediated expression of VEGF and insufficient surfactant production (73). Moreover, a similar phenotype is induced in mice by specifically deleting the HRE located within the *Vegf* promoter. Targeted deletion of ARNT, the dimerization partner for both HIF-1α and HIF-2α, as well as for other transcription factors, also results in embryonic lethality at E10.5, with affected embryos displaying defective angiogenesis of the yolk sac and branchial arteries (74).

Experimental studies in animal models of BPD suggest that HIF family members are important for late lung development in general and, in specific, that HIF plays an important role in both normal pulmonary vascular development and abnormal pulmonary vascular remodeling. HIF-1α and HIF-2α protein are decreased in the lungs of preterm baboon and lambs undergoing mechanical ventilation (69, 75). Expression of HIF-2α is also decreased in the lungs of neonatal rats exposed to chronic hypoxia, another stimulus that impairs alveolar development and decreases pulmonary vascular growth in mice (76). Enhancement of HIF signaling by either selective or non-selective inhibition of PHD-mediated HIF degradation increases angiogenesis of lung microvascular endothelial cells *in vitro*, in association with increases in PECAM-1, VEGF, and FLT-1 (77). A similar strategy to stabilize HIFs *in vivo* increases VEGF and PECAM expression in the lungs of preterm baboons (78), and improves alveolarization, oxygenation, and lung compliance (79). In a newborn rat model of BPD induced by intra-amniotic LPS followed by hyperoxia, non-selective inhibition of PHDs stabilizes HIF-1α in the whole lung, and attenuates the disrupted alveolar and vascular growth observed in this model (80). Interestingly, sildenafil, a phosphodiesterase inhibitor that has been used clinically to treat PH by increasing cGMP levels, improves alveolarization in neonatal mice exposed to hyperoxia and directly activates HIF-1α-mediated signaling in airway epithelial cells (81).

### Nitric Oxide

Nitric oxide (NO) is a free radical gas that functions as a second messenger, regulating diverse physiologic processes such as angiogenesis, vasodilation, and anticoagulation (82). NO is produced by the nitric oxide synthase (NOS) family of proteins, which contains three isoforms: neuronal NOS (*NOS1*), inducible NOS (*NOS2*), and endothelial NOS (*NOS3*). After release from the endothelium, NO can diffuse to the luminal side of the vessel to inhibit platelet aggregation and adhesion or to the abluminal side of the vessel where it regulates vascular smooth muscle contraction and proliferation (83). Many of the downstream effects of NO on vascular tone result from the ability of NO to activate soluble guanylyl cyclase, thereby increasing cGMP and decreasing intracellular calcium.

Endothelial nitric oxide synthase (eNOS), initially believed to be expressed solely by endothelial cells in a constitutive fashion, is now known to be expressed by additional cell types (84) and dynamically regulated in response to hypoxia, inflammation, and other factors (84, 85). Importantly, VEGF induces eNOS expression *via* a FLK-1-dependent mechanism (86, 87), and loss of eNOS impairs VEGF-mediated angiogenesis (88). NO is a downstream effector of VEGF-mediated angiogenesis but not fibroblast growth factor (FGF)-mediated angiogenesis (89), and *eNOS*^−/−^ mice demonstrate impaired VEGF-mediated angiogenesis (88) and neovascularization during wound healing and after ischemia (90, 91). Expression of eNOS is modulated by changes in oxygen tension both *in vitro* and *in vivo*. NOS activity in pulmonary artery endothelial cells increases at higher oxygen concentrations and decreases at lower oxygen concentrations (92), an effect mediated by both transcriptional and posttranscriptional mechanisms (93).

Decreased expression of eNOS is observed in a number of animal models of BPD. Chronic ventilation of preterm lambs increases pulmonary vascular and airway resistance, and decreases eNOS protein expression in the endothelium of the small intrapulmonary arteries and the airway epithelium (94). Similarly, chronic ventilation of extremely preterm fetal baboons also decreases lung eNOS expression (95). Intra-amniotic endotoxin also decreases eNOS expression in the lungs of fetal lambs, particularly in small pulmonary arteries (96). In an ovine model, intrauterine growth restriction decreases pulmonary vascular density and alveolarization, in association with decreases in VEGF-induced NO production in large proximal pulmonary arteries (97).

In adult mice, compensatory lung growth after pneumonectomy is severely impaired by targeted deletion of eNOS or inhibition of NO production with a NOS inhibitor (98). Exposing adult *eNOS*^−/−^ mice to mild hypoxia induces more severe PH than that seen in control mice (99), and exposing neonatal *eNOS*^−/−^ mice to mild hypoxia impairs alveolarization and decreases pulmonary vascular density (100). In both models, these detrimental effects on pulmonary pressures and lung structure are rescued by iNO (99, 101). Further, iNO appears to have beneficial effect in other experimental models of BPD. Treatment of neonatal rats with a single dose of the FLK-1 inhibitor, SU-5416, impairs alveolarization and induces RVH, and iNO administration prevents RVH development and significantly increases radial alveolar counts (102). Prolonged iNO therapy also prevents RVH and partially rescues the severe defect in alveolarization induced by bleomycin in neonatal rats (103).

## Additional Molecular Mechanisms That may Influence Alveolar and Vascular Growth

In addition to the well-established molecular pathways described above that are central regulators of normal pulmonary vascular development and function, a number of additional molecules and pathways have been recently identified that also appear play a role in the aberrant vascular growth observed in BPD.

### Hydrogen Sulfide

In addition to NO, hydrogen sulfide (H_2_S) is an additional gasotransmitter that appears to have an important role in late lung development. H_2_S is produced by two main enzymes: cystathionine β-synthase (Cbs) and cystathionine γ-lysase (Cth). Deletion of either *Cbs* or *Cth* decreases alveolar number by 50%, reduces the pulmonary vascular supply, and increases the number of muscularized small and medium-sized pulmonary arteries (104). In addition, H_2_S appears to have important, direct effects on the angiogenic function of pulmonary endothelial cells. Silencing or pharmacologic inhibition of Cbs and Cth, respectively, impairs *in vitro* tube formation in human lung endothelial cells, and conversely, exogenous administration of H_2_S enhances tube formation *in vitro* (104). Further, exogenous administration of H_2_S improves alveolarization *in vivo* and limits PH in hyperoxia-exposed neonatal rats (105); and improves epithelial repair and decreases inflammation in hyperoxia-exposed neonatal mice (106).

### Retinoic Acid

Retinoic acid (RA) is a biologically active derivative of vitamin A. Early studies identified a role for vitamin A and RA in enhancing limb regeneration in amphibians after amputation (107). Subsequently, RA was shown to promote alveolar regeneration in adult rats in elastase-induced emphysema (108) and to blunt the impaired alveolarization induced by dexamethasone in neonatal rats (109). Mice with genetic deletion in the RA-receptor-gamma have decreased lung elastin and impaired alveolarization (110). Pulmonary endothelial cells are a source of RA in the developing lung, where it appears to promote pulmonary angiogenesis by increasing the expression of VEGF-A and to regulate elastin synthesis by increasing FGF-18 expression (111).

### Lysophosphatidic Acid

Lysophosphatidic acid is a small glycerophospholipid that exerts multiple biologic effects on cell proliferation, migration, survival, and cell–cell interactions by binding to G-protein coupled receptors on the cell membrane (112). LPA appears to have an important role in many lung diseases, functioning to regulate airway inflammation, remodeling, and fibrosis (113–115). In the vasculature, LPA can function as either a vasodilator or a vasopressor depending on context. For example, in the thoracic aorta, LPA causes NOS-dependent vasodilation by acting through the LPA receptor-1 (LPAR1). Mice containing mutations in the LPAR1 demonstrate decreased lung inflammation and fibrosis and improved survival in an experimental model of BPD, and pharmacologic blockade of the LPAR-1 and -3 protects against pathologic vascular remodeling, limiting muscularization and RVH in newborn rats exposed to chronic hyperoxia (116). Although there were some phenotypic differences between the mice with genetic deletions of LPAR-1 and pharmacologic blockade that require future study, these studies suggest that the LPA pathway may prove to be a promising new target for BPD.

### Extracellular Superoxide Dismutase

Extracellular superoxide dismutase (EC-SOD) is a potent antioxidant that catalyzes the dismutation of superoxide to hydrogen peroxide and oxygen (117). EC-SOD is highly expressed in the lung and vasculature, and EC-SOD expression and activity is suppressed in experimental models of BPD (118). Alveolar epithelial overexpression of EC-SOD preserves alveolar surface and volume density, decreases inflammation in newborn mice exposed to hyperoxia (119), and attenuates pathologic vascular remodeling and PH in adult mice exposed to chronic hypoxia (120). Conversely, deletion of EC-SOD impairs alveolarization in neonatal mice and decreases pulmonary vascular density and Flk-1 protein expression (118). Taken together, these studies highlight the importance of tight control of the oxidative balance in the lung in promoting physiologic alveolar and vascular growth, and preventing pathologic airway and vascular remodeling.

### Stem and Progenitor Cells

A number of resident stem and progenitor cell populations have been identified in the lung, deriving from epithelial, mesenchymal, and endothelial origins. Each population is unique in its defining characteristics and putative functions, which are comprehensively discussed in a number of excellent, recent reviews (121–123). Accumulating evidence from clinical and experimental studies have suggested that alterations in circulating and/or resident lung stem and progenitor cells may contribute to the pathogenesis of BPD, sparking great interest in the investigation of cell-based therapeutic strategies as a potential treatment for BPD. Hyperoxia decreases lung and circulating endothelial progenitor cells in neonatal mice (124), and diminishes the number of lung side population (SP) progenitor cells, a population believed to have both epithelial and mesenchymal potential (125). Further, studies in experimental models suggest that mesenchymal stem cell therapy may have beneficial effects on preserving alveolar and vascular growth during injury. Intratracheal administration of mesenchymal stem cells attenuates induced lung cell apoptosis and inflammation, and improves alveolarization in neonatal rats exposed to hyperoxia (126). Intravenous administration of bone marrow-derived mesenchymal stem cells (BMSCs) in neonatal mice prevents PH and blunts the impaired alveolarization induced by hyperoxia despite a low level of engraftment. Importantly, in that study, the administration of conditioned media of these stem cells had an even greater beneficial effect, preserving normal alveolarization and preventing pathologic vascular remodeling (127). A similar improvement in alveolar and vascular growth is observed in hyperoxia-exposed neonatal rats after intratracheal administration of BMSCs (128), and this beneficial effect is evident even if the MSCs are administered after the initiation of lung injury (129). Moreover, MSC treatment results in durable improvements in lung structure, with sustained improvement in lung structure and exercise tolerance in adult mice at 6 months of age, and an absence of any evidence of long-term detrimental side effects. These exciting data prompted clinical studies to assess whether alterations in lung progenitor cells play a role in BPD, discussed in the following section.

## Alterations in Angiogenic Pathways in Patients with Bronchopulmonary Dysplasia

These data, obtained from experimental models demonstrating disruption of key pathways known to promote physiologic pulmonary angiogenesis, appear to have some fidelity with the human disease. The impaired pulmonary vascular development observed in infants dying of severe BPD is associated with decreased expression of VEGF and FLT-1 (6). In response to short-term ventilation, the expression of classic angiogenic growth factors, such as VEGF and angiopoietin-1, decreases in the lungs of preterm infants, while expression of endoglin increases, suggesting that endoglin may be one important regulator of the vascular remodeling which occurs in BPD (130). In a similar, but separate, study by the same group, short-term ventilation decreases the gene expression of proangiogenic factors such as *FLK-1*, TEK tryrosine kinase, endothelial (*TIE-2)*, and angiogenin, yet increases the expression of anti-angiogenic mediators such as thrombospondin-1 (131). Taken together, these two studies suggest that even short-term mechanical ventilation causes widespread alterations in a variety of angiogenic signaling pathways in the developing lung.

In contrast to these studies demonstrating changes in the gene and protein expression of angiogenic mediators from whole lung tissue of patients dying with BPD, studies evaluating levels of VEGF in the tracheal fluid have not shown clear differences between preterm infants who develop and those who do not develop BPD. Lassus et al. found that the levels of VEGF in tracheal fluid obtained during the first 10 days of life are not significantly different in preterm infants who developed BPD versus those who do not develop BPD (132). In keeping with these results, two additional studies demonstrated that tracheal fluid VEGF levels obtained during the first month of life also did not correlate with the development of BPD (133). However, it is not clear whether the absence of positive findings in these studies represent differences between the pathogenesis of experimental BPD and the human disease, a lack of statistical power, or the inability of tracheal aspirates to reflect the true microenvironment present in the developing lung.

Similarly, despite strong experimental evidence demonstrating the importance of both the HIF and NO signaling pathways in physiologic pulmonary angiogenesis, data assessing the integrity of the HIF of NO signaling in patients with BPD remain scarce. In the developing human lung, both *HIF-2α* and *VEGFA* gene expression demonstrate a positive correlation as lung development progresses; however, little is known regarding how HIF activity or expression is altered in preterm infants with BPD. Similarly, there is an absence of data directly demonstrating decreased NOS expression or NO production in infants with BPD. However, the levels of the endogenous NOS inhibitor, asymmetric dimethylarginine (ADMA), are increased in patients with BPD and PH, suggesting that heightened levels of ADMA may contribute to the increased PVR observed in patients with BPD and PH by limiting NO production (134). Yet, despite extensive experimental evidence demonstrating disruptions in NO signaling and the therapeutic benefit of iNO therapy in animal models, a number of recent, prospective, and randomized trials have failed to demonstrate beneficial effects of iNO therapy in the prevention of BPD in preterm infants (135–137).

Given the accumulating evidence from experimental models that demonstrated the beneficial role of stem and progenitor cells in promoting alveolar and vascular growth during injury, clinical studies aimed to determine whether disruption of angiogenic progenitors might contribute to the pathophysiology of BPD. Late outgrowth endothelial colony-forming cells (ECFCs), a sub-type of EPCs that are highly proliferative, self-renewing, and capable of forming blood vessels *de novo in vivo* (138). ECFCs obtained from preterm infants are more proliferative than those obtained from term infants, yet more highly susceptible to the growth inhibiting effects of hyperoxia (139). In a small, early prospective study, ECFCs were found to be low in extremely premature infants and to increase with increasing gestation. Further, extremely preterm infants with lower numbers of ECFC were found to be at increased risk of developing BPD (140). In keeping with these results, a subsequent study confirmed that cord blood ECFCs are significantly lower in preterm infants who go onto develop moderate or severe BPD (141). Taken together, these studies lend further support to the notion that antenatal events may influence later respiratory outcomes, and suggest that ECFC may represent a biomarker for the identification of patients at greatest risk for the development of BPD. In addition to these endothelial progenitors, another small clinical study demonstrated the presence of fibroblast-like cells with colony-forming potential and cell surface marked similar to MSCs in the tracheal aspirates of premature infants with RDS. After adjusting for numerous potential confounders, including gestational age, duration of mechanical ventilation, and others, the presence of these tracheal MSC predicted the development of BPD (142). Although clinical evidence regarding the role of MSC in patients with BPD is limited, the strong experimental evidence demonstrating the benefit of MSC therapy on alveolar and vascular growth in animal models has already lead the way for phase 1 clinical trails for testing this therapy in preterm infants at high risk for BPD (143).

## Conclusion

Over the past three decades, significant advances in the supportive care of extremely premature infants, including surfactant replacement therapy, have significantly decreased mortality from BPD, yet, the morbidity associated with BPD remains high. Numerous abnormalities of the pulmonary circulation are observed in patients with BPD, influencing long-term prognosis, including dysmorphic pulmonary capillary development, maladaptive pulmonary vascular remodeling, heighted pulmonary vascular tone, and the development of abnormal collateral circulation. Extensive experimental and clinical data derived form studies over the last decade have advanced our understanding of the pathobiology contributing to BPD, including the recognition that pulmonary angiogenesis is essential for alveolarization, and that disrupted pulmonary angiogenesis likely contributes to BPD. Given the limited availability of human lung tissue from patients with BPD, much of our understanding of the molecular mechanisms involved have been derived from experimental animal models (144), and definitive clinical evidence demonstrating that these same mechanisms are causative in the human disease are lacking. Nonetheless, these studies suggest that replacement of angiogenic factors and/or stem cell-based therapies could prove to be beneficial for the treatment of BPD. Moving forward, the development of innovative non-invasive diagnostic technologies that may permit an accurate assessment of the molecular pathways that are dysregulated in patients at risk for BPD will be required in order to foster the development of targeted biologic therapies that can effectively stimulate lung growth and regeneration.

## Author Contributions

Dr. CA composed the manuscript and designed the figure and table.

## Conflict of Interest Statement

The author declares that the research was conducted in the absence of any commercial or financial relationships that could be construed as a potential conflict of interest. The Guest Associate Editor AH declares that, despite having collaborated with the author CA, the review process was handled objectively.
